# Connect or detach: A transformative experience for medical students in end‐of‐life care

**DOI:** 10.1111/medu.15545

**Published:** 2024-09-24

**Authors:** Diego Lima Ribeiro, Daniele Sacardo, Grazyna Drzazga, Marco Antonio de Carvalho‐Filho

**Affiliations:** ^1^ Faculty of Medical Sciences University of Campinas Campinas Brazil; ^2^ University Medical Center Groningen University of Groningen Groningen The Netherlands; ^3^ Department Public Health University of Campinas Campinas Brazil; ^4^ Lifelong Learning, Education, and Assessment Research Network (LEARN) University of Groningen Groningen The Netherlands; ^5^ Wenckebach Institute (WIOO), Lifelong Learning, Education, and Assessment Research Network (LEARN) University Medical Center Groningen Groningen The Netherlands

## Abstract

**Context:**

At the beginning of clinical practice, medical students face complex end‐of‐life (EoL) decisions, such as limiting life‐sustaining therapies, which may precipitate emotionally charged moral dilemmas. Previous research shows these dilemmas may cause identity dissonance and impact students' personal and professional development. Despite the prevalence of such dilemmas, medical educators have limited insight into how students navigate these often emotional experiences. This study explores how medical students make sense of and deal with moral dilemmas lived during EoL's care.

**Methods:**

This cross‐sectional qualitative study used thematic analysis (Braun and Clarke) to analyse interviews with 11 Brazilian final‐year medical students. The interviews followed the drawing of a rich picture representing moral dilemmas experienced by medical students when engaging with EoL care. The reporting of this study follows the Standards for Reporting Qualitative Research (SRQR).

**Results:**

Participants highlighted four main themes when engaging with EoL care: ‘experiencing death’, ‘making decisions at the end‐of‐life’, ‘connecting versus detaching: an upsetting dilemma’ and ‘being transformed’. They described the emotional overwhelm of experiencing death and the uncertainty in navigating EoL decisions. The central moral dilemma faced was whether to connect with or detach from patients. This dilemma was lived in the context of a hidden curriculum that preaches emotional distancing as a coping mechanism. Developing the moral courage to overcome this barrier and choosing to connect became a transformative experience, significantly impacting their personal and professional development and reinforcing their commitment to patient‐centred care.

**Conclusion:**

Connecting with patients in EoL care involves breaking cultural norms to establish meaningful connections with patients aiming for compassionate care. This process may lead to identity dissonance and also represents an opportunity for transformative learning. Educators can support this transformative process by legitimating students' connections with patients, teaching emotional regulation strategies, and leveraging personal experiences to foster trust.

## Abstract 2

Exploring emotional dilemmas associated with end‐of‐life decisions, @diegolimaribei4 reveal how final year medical students facing the pivotal moral challenge of connecting with or detaching from patients is a transformative journey.

## 1 INTRODUCTION

As medical students transition into the realm of clinical practice, they actively engage in intricate end‐of‐life (EoL) decisions that often involve limiting life‐sustaining therapies.^1^, ^2^, ^3^, ^4^ Participating in such complex decisions may give rise to emotionally charged moral dilemmas.^5^, ^6^ Medical educators still have limited insight into how students navigate these dilemmas, manage their emotional reactions, and make sense of these experiences.^7^ Furthermore, medical educators still do not fully recognise how engaging with these dilemmas impacts medical students' journey to become a doctor and develop a professional identity. This study seeks to unravel the intricacies of medical students' moral dilemmas in EoL care decision‐making. We believe that understanding how students deal with and cope with moral dilemmas in EoL decision‐making can aid educators in designing targeted learning activities to nurture students' professional identity formation.

Once medical students begin their clinical practice and engage with patient care, they often participate in EoL care decisions regarding withholding or withdrawing life‐sustaining therapy.^1^, ^8^, ^9^ Withholding means not starting new therapeutic actions beyond the ones already in place.^10^, ^11^ For instance, not introducing vasoactive drugs for a patient with hypoperfusion in the context of inevitable death.^12^, ^13^, ^14^ Withdrawing refers to stopping a treatment initially intended to sustain life,^10^, ^11^ for instance, suspending mechanical ventilation and removing the orotracheal tube from a patient who is deteriorating progressively despite optimal clinical care.^13^, ^14^ Withholding and withdrawing life‐sustaining therapies are common in the context of EoL care when health‐care providers recognise that continuing life‐sustaining therapies would only prolong the patient's suffering and the dying process.^15^, ^16^ These complex therapeutic decisions may trigger challenging moral dilemmas especially in health‐care providers without relevant clinical experience in EoL, such as medical students.^14^, ^17^, ^18^, ^19^

In a previous study, we explored how medical students' moral dilemmas trigger powerful emotional reactions that influence professional identity formation at the beginning of clinical training.^20^ We define a moral dilemma as a situation in which students deal with conflicting values, where the available options endorse mutually inconsistent lines of action, and none of the options seem sufficient.^20^, ^21^ In the aforementioned study, we elaborated on how engaging with moral dilemmas evokes medical students' intense and long‐lasting emotional responses, such as frustration, anger and anxiety.^20^ We described how navigating this emotional turmoil toward conflicting values may result in identity dissonance. Costello described identity dissonance as experiences in which students go through significant distress while attempting to reconcile conflicting elements of the professional role with their former identities.^22^ Developing a dissonant identity may prompt students to reevaluate their values, aspirations and abilities, which can result in emotional suffering and consideration of abandoning the course.^23^, ^24^, ^25^, ^26^

Based on our previous findings and the complexity of EoL care, we believe that facing moral dilemmas in the context of withholding and withdrawing life‐sustaining therapies can evoke powerful emotional responses in medical students, potentially leading to identity dissonance. Understanding the impact of these dilemmas on students' professional development is crucial for tailoring pedagogical strategies to support students' professional identity formation. In this study, we explore how medical students make sense, cope and deal with moral dilemmas during therapeutic withdrawal/withholding decisions amid EoL care.

## 2 METHODS

### 2.1 Design

We conducted a cross‐sectional qualitative study, adopting a constructivist paradigm,^27^, ^28^, ^29^ which acknowledges reality as an active construction emerging from the interaction of the researcher with the world. We used thematic analysis^30^, ^31^ as our guiding framework, which directed the gathering and examination of a dataset of rich pictures^32^, ^33^ and individual interviews with final‐year medical students.^33^ We collected and analysed the data iteratively, ensuring a dynamic interplay between data collection and data analysis. This study received ethical approval from the research ethics committee of the State University of Campinas (CAAE: 55505121.2.0000.5404).

### 2.2 Context

We carried out this study at a Brazilian medical school. In Brazil, the medical curriculum spans 6 years, with the final 2 years, involving daily clinical activities, often with students taking a prominent role. It is noteworthy that newly graduated Brazilian physicians can practice autonomously in primary and emergency care without additional residency training, which justifies this early engagement in intense clinical practice. We conducted interviews with students during their sixth year, ensuring they had gained at least 1 year of hands‐on, in‐hospital clinical experience—a year during which they progressively became more autonomous.

Palliative care in Brazil faces significant challenges that impact the educational context of our study. Awareness and understanding of palliative care are often limited, with many misconceiving it as solely end‐of‐life care. Despite the establishment of the National Policy for Palliative Care in 2018, implementation remains inconsistent because of a lack of specific funding and regulatory frameworks. Furthermore, cultural attitudes towards death, influenced by religious beliefs and societal norms, may complicate and delay the adoption of palliative care in some medical settings.

### 2.3 Participants

We purposefully recruited students who have experienced moral dilemmas related to withdrawing/withholding decisions in the context of EoL care by making it clear on the invitation for the study. We invited students from diverse internship groups, genders and ages to ensure comprehensive representation. DLR, the main author, approached students' groups during their clinical rotations, describing the research objectives and methodology. Interested students then approached DLR to schedule interviews, allowing us to identify and engage students who were motivated and willing to candidly share their experiences. On the agreed‐upon day, the students signed the consent form and participated in the rich picture/interview session.

### 2.4 Rich pictures

The ‘rich picture’ is a visual approach rooted in systems engineering that leverages visual narratives to encapsulate an individual's multifaceted perspective on a given situation, encompassing tangible elements, abstract concepts, people, characters, emotions and interpersonal conflicts.^33^, ^34^ This method prompts participants to approach their experiential narratives from fresh and novel viewpoints and articulate thoughts that often are difficult to verbalise, allowing new insights.^32^ In the realm of medical education research, rich pictures methodology has been used to delve into complex situations,^20^, ^32^ including understanding emotional reactions and motivation of trainees,^34^, ^35^ medical expert judgements^36^ and learning in the clinical environment.^37^

Considering the inherent emotional complexity that underlies end‐of‐life moral dilemmas, we hypothesised that rich pictures would complement traditional interviews by allowing participants to deepen their reflections and elucidate the emotional and non‐verbal dimensions of their experiences. Additionally, the act of drawing the rich picture provides an opportunity for introspection. This thoughtful process of visualising complex lived situations often culminates in cathartic moments during the interviews, when participants express their emotions through crying, slapping the table or expressing tear‐filled eyes with a serene smile.

### 2.5 Data collection

First, DLR instructed the participants to draw a real end‐of‐life situation that happened during a clinical rotation in which they experienced a moral dilemma related to withdrawal or withholding life‐sustaining therapies. The participants were provided with definitions of key terms (Appendix S1) and reassured about the voluntary nature of their participation through a detailed informed consent process. This process emphasised their right to withdraw at any time without affecting their academic status or relationships. Interviews were conducted in private settings to ensure confidentiality and uphold ethical standards. Subsequently, the participants were left alone in an exclusive, private room for 20 min. They were provided with a sheet of A3 paper and a selection of grey and coloured pencils and markers. The subsequent phase involved conducting interviews, adhering to a structured five‐step protocol (Appendix S1). A total of 11 students joined the study, five females and six males, with an average age of 23.9 years. The sessions lasted an average of 1 h (total 11h03min), averaging 19 min per drawing (total 3h36min) and 40 min per interview (total 7h27min).

### 2.6 Sampling strategy

Our sampling strategy was guided by the concept of information power.^38^, ^39^ Information power is achieved when the data generated through the analysis and theoretical interpretation of the pictures and insights garnered from students' interviews furnished us with sufficient information to construct novel knowledge and effectively address our research inquiries.^38^ In essence, our data collection ceased after 11 interviews, when we had gained a comprehensive understanding of the intricate facets of students' emotional responses, internal negotiation processes, response patterns and the transformative impact of their experiences.

### 2.7 Data analysis

DLR was responsible for collecting the data. DLR, MACF and DS performed a thematic analysis in six steps as described by Braun and Clarke.^31^ Throughout the analysis, they iteratively and constantly compared new data and interpretations with previous assumptions and understandings. In this way, the interviews and rich pictures were analysed in parallel, with the analysis of one informing the analysis of the other in a constant iterative process. To facilitate understanding, we will describe the analysis of the interviews and rich pictures separately.

Interviews Analysis – First, DLR, DS and MACF independently read and re‐read the first three transcripts. Second, DLR and DS developed an initial coding guided by the research question: ‘how medical students make sense, cope, and deal with moral dilemmas during therapeutic withdrawal/withholding decisions amid EoL care?’ They met weekly to compare the codes in a process that allowed the addition, subtraction or transformation of codes. MACF joined this discussion to enhance the understanding of the codes and identify gaps in the data that required additional exploration. Third, the authors searched for themes, comparing their interpretations, and contrasting assumptions and understandings to reach a consensus on the initial themes. Fourth, the research team reflected on the first three interviews analysis and elaborated on the topics to address in the following interviews. While reflecting on the first interviews, the research team realised that witnessing the death process and participating in EoL care decision‐making was remarkable for the students. Thus, in the following interviews, DLR (a) invited students to deepen their reflections (already started with the drawing) on experiencing death, searching for the emotional reactions, behaviours responses, and meanings; and (b) explored how EoL experiences could influence students' personal life and professional development. DLR was thorough in keeping the conversation open, adopting an active listening attitude and constantly elaborating on the following question from the perspective of the last answer. Fifth, whenever deemed essential, the research team held meaningful and insightful deliberations to expand upon the constituent elements, context, mechanisms, and relationships surrounding students' experiences and moral dilemmas. This allowed them to create a coherent and compelling narrative that explained the themes and their relationships. Sixth, the research team collaborated in the writing of the manuscript. DLR recorded the entire process of coding and developing themes and mind maps in a logbook. Furthermore, the research team meetings were reflective spaces where researchers could check the validity of findings. For the two last steps (fifth and sixth), GD joined the group bringing a complementary perspective that helped the group to refine the narrative and, as an outsider, to provide new insights and construct new meanings. This final stage involved meticulous documentation of the research process, adhering to the Standards for Reporting Qualitative Research (SRQR)^40^ to ensure thorough and transparent reporting.

*Rich pictures analysis* – DLR, MACF and DS analysed the drawings in group sessions. These group sessions adhered to a standardised structure.^33^ First, DLR presented the drawing to the researchers unfamiliar with the interview's content and the researchers engaged in a dialogical discourse focussing on an initial, non‐interpretative description of the pictorial representation, paying particular attention to spatial arrangement, use of colours, metaphorical elements and symbolic depictions. Second, the researchers explored potential interpretations of the drawing's elements, trying to decipher the underlying messages. Third, DLR shared the story behind the drawing and the researchers engaged in a meaning‐making exercise based on the research question.^41^ The group tried to identify the moral dilemmas at hand, its constituent elements, the implied values, the main characters, and students' emotional responses and discern the potential impacts on their professional development. Each session lasted around 1h and was taped by DLR, who kept a logbook. This logbook served as an invaluable instrument to preserve the reflective process, facilitate later cross‐referencing with the transcribed interviews, and provide an overarching framework to maintain continuity in the data analysis. The data analysis and collection proceeded in iterative cycles adhering to the following sequence: analysing the drawings, transcribing and coding of new interviews, refining of the interview protocol, coding of the interview transcripts, re‐checking interviews and pictures, and further enhancing of the process of developing codes and themes.

### 2.8 Research group and reflexivity

Our research team comprised individuals with diverse and complementary expertise deemed pertinent to the data analysis. DLR is a general clinical practitioner with experience in undergraduate and postgraduate medical education who worked as an assistant physician in the emergency room, internal medicine ward and intensive care unit in the same institution where data were collected. He brought an insider's perspective, enriched by his own encounters with EoL decision‐making and the dying process. DS is a psychologist and bioethics professor in the same institution with a particular interest in identity and moral development. GD, a cognitive linguist with a background in medical education and faculty development, brings a unique blend of professional and cultural perspectives to the table. With expertise in qualitative research, GD's outsider status has provided the research team with a valuable opportunity to question commonly accepted assumptions and reinterpret familiar concepts in a manner that is more comprehensible to the general public. DS and GD brought a non‐clinical viewpoint, which played a crucial role in assisting DLR and MACF in adopting alternative perspectives, challenging assumptions that were otherwise accepted as natural or culturally ingrained. Finally, MACF is a full professor of Health Profession Education Research, who served as a clinical teacher for 15 years in the same institution where the data were collected. MACF has experience with EoL care and his research focus lies in the transition of medical students into professional practice. MACF's wealth of experience with terminally ill patients in challenging contexts enabled him to bridge the clinical and educational perspectives.

## 3 RESULTS

The participants in our study articulated their experiences through four main themes that we elaborated from the data: ‘experiencing death’, ‘making decisions at the EoL’, ‘connecting versus detaching: an upsetting dilemma’ and ‘being transformed’. Each theme captures a critical aspect of the medical students' engagement with EoL care. The participants stressed how experiencing death was emotionally overwhelming and how uncertain and ambiguous it was to navigate EoL decision‐making processes. The emotional charge caused by the death experience, associated with the cognitive overload of engaging with the decision‐making, created the realm for the main moral dilemma participants experienced: connecting with patients or detaching from them. Managing this dilemma was a transformative experience that impacted students' personal and professional development. In the following sections, we will elaborate on each theme in detail.

### 3.1 Experiencing death

Experiencing death constitutes a multifaceted and emotionally charged experience for students, involving (1) witnessing patients' deaths and (2) navigating a medical culture that often sidesteps open discussions about death and dying.

During their clinical training, medical students encounter a profoundly impactful moment the first time when they witness death. This moment marks their initial recognition that death is often not a singular moment in time or a precise instant but a process that can span hours, days or even weeks. They feel shocked when they are simultaneously confronted by death's inexorable progression and pronounced physical changes, such as intense edema, loss of eye brightness, cyanosis and severe malnutrition, among others. While taking students by surprise, these physical changes cause extreme discomfort and awaken, almost involuntary, feelings of disgust and repulsion. When talking about these feelings, students openly expressed shame, which remained present during some of the interviews.

**RPS8** *‘I think I felt… it's not a pleasant feeling, but I guess I felt disgusted. Not because of her, but it (the situation) was very morbid. The lower limbs were very morbid. The first feeling was: “Wow, how sick she is”. Because from the trunk upwards, it was sarcopenic, and from the neck downwards, it was extremely edematous.’*

**FIGURE 1:**
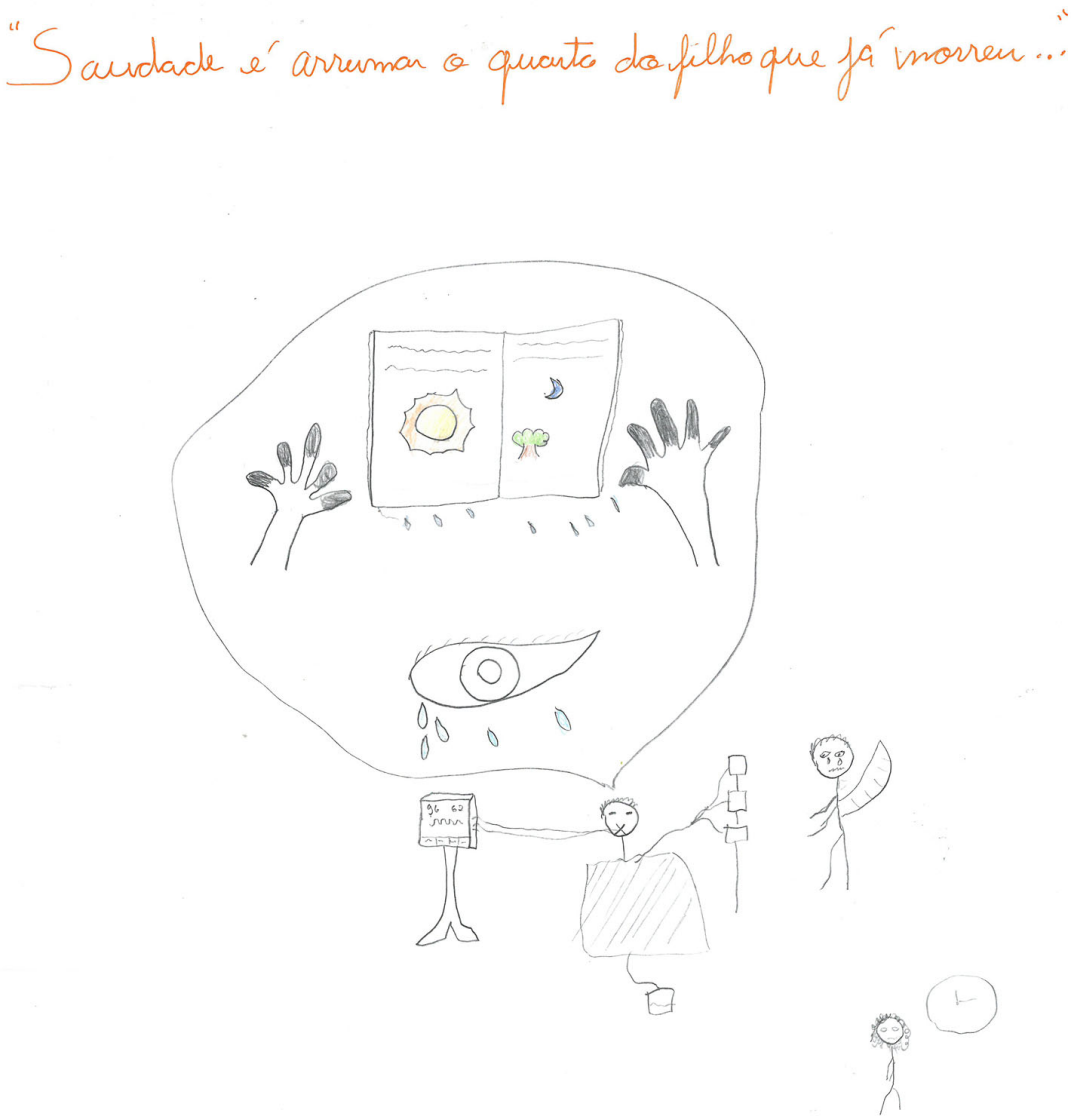
‘Longing is tidying up the room of a son who has already passed away.’ (rich picture 3—RPS3/State University of Campinas, 2022–2023): The student (Clara—fictitious name) shared a profound experience lived in the emergency room during her emergency medicine rotation. Marcia (patient's fictitious name) was a 40‐year‐old woman with lifelong physical and cognitive limitations resulting from neonatal hypoxia. Marcia's hospitalisation was because of septic shock that ultimately led to multiple organ dysfunction. When Clara met Marcia, she was reliant on an orotracheal tube, ventilatory support and high doses of noradrenaline, although these efforts were unsuccessful in stabilising her blood pressure. Clara was deeply struck by the pronounced cyanosis in Marcia's limbs and ears, as well as the fading brightness in her eyes. Clara also delved into Marcia's life story through conversations with her mother and brother. She learned that Marcia had bravely overcome a lifetime of bullying related to her physical limitations and that she was an aspiring writer. Marcia's hospitalisation had left her last book unfinished. As Clara witnessed the inexorable progression of the disease, she closely followed the emotional anguish experienced by Marcia's mother and brother, especially when they, alongside the medical team, had to make the heart‐wrenching decision to withdraw life‐sustaining therapy. Marcia passed away just hours after this difficult choice. The picture's title refers to the grief of Marcia's mother over her daughter's death and is a citation of a famous Brazilian song about mourning. In the centre of the drawing, Clara represents Marcia, with a balloon comprising her cyanotic hands, her dull eyes and the unfinished book. To the right and below, Clara draws herself with a clock signifying her reflection on her own life and the realisation that it, too, would 1 day come to an end. She has portrayed the patient as larger and centrally positioned in the drawing to represent that the patient should always remain at the core of health care. [Color figure can be viewed at wileyonlinelibrary.com]

While observing the physical aspects of a patient's deterioration, the students also witness a phenomenon they termed ‘depersonalisation’—a gradual decline in a patient's ability to carry out daily activities and maintain close, meaningful relationships with family and friends. This depersonalisation extends beyond the mere physical ailments a patient may be facing, delving into the profound impact of patients' medical condition on their overall quality of life. In other words, students beheld with a heavy heart the person slowly falling apart from who they were, even before their evident physical breakdown (Figure 1). This close contact with death and dying reminded students of their own mortality, raising feelings of sadness, sorrow and fear of experiencing a similar process in the future.

**Researcher:** *‘You said you drew her eye without color. Why?’*

**RPS3:** *‘I think it represents a glimpse of the end, where we are losing, losing life, losing our ability to do the things we once did. The person becomes depersonalized. We don't even know the color of the eye anymore.’*

The challenges of dealing with the death experience were intensified by a workplace environment where death is a taboo and the primary coping mechanism is based on emotional detachment. Within this context, students reported living in a culture where dying is not acknowledged by the care team, including medical residents and supervisors. Despite anticipated poor outcomes, the patients continue to undergo life‐sustaining therapies such as orotracheal intubation, mechanical ventilation and haemodialysis. Students reported situations where doctors initiated or maintained those treatments even without knowledge of the patients' and their families' wishes. In this environment, students often felt discouraged from discussing death or forming strong bonds with their patients. Moreover, students reported that, when advocating for EoL care, supervisors who were not familiar with palliative care concepts frequently dismissed their input, stating they did not have enough experience or knowledge. This led to doubts about their competencies and reasoning skills. Those doubts and intense feelings of anguish and insecurity were remarkable during the EoL decision‐making process.

**RPS9:** *‘I think every doctor thinks they understand palliative care, but they don't. Upon graduation, we (students) are also not encouraged to understand palliative care. People are encouraged to treat. And when you get to that stage, when there is nothing left to treat, but you can still provide care, I sometimes feel that it triggers anguish in a doctor because they don't know what to do.’*

### 3.2 Making decisions at the EoL

**FIGURE 2:**
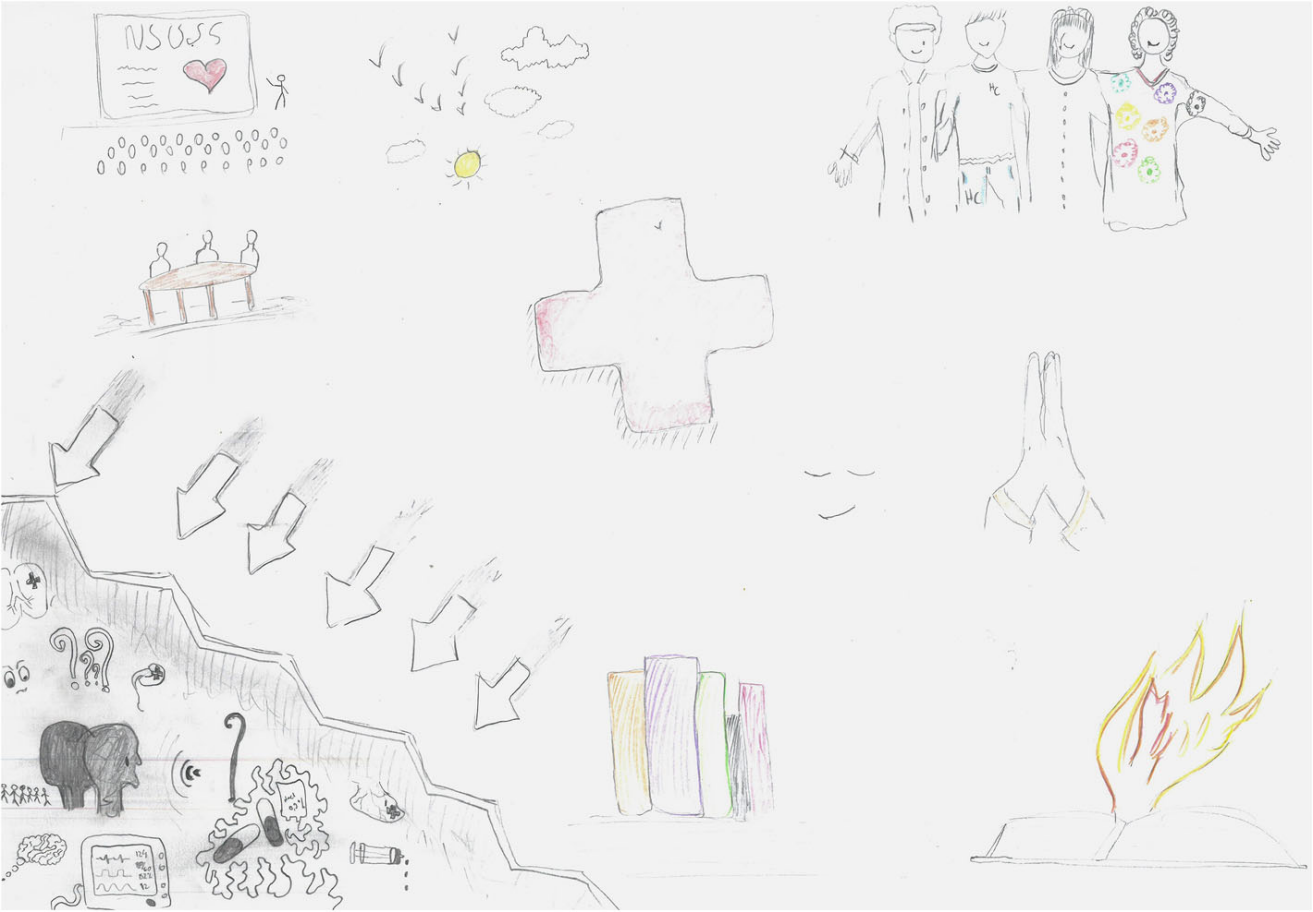
No title (rich picture 7—RPS7/State University of Campinas, 2022–2023): Pedro (fictitious name) tells the story of his experience during his intensive care internship involving a patient named Carlos (fictitious name). Carlos had advanced gastric cancer and was admitted to the intensive care unit because of deteriorating septic shock, despite extensive life‐sustaining treatments like mechanical ventilation, vasoactive medications and haemodialysis. As the health‐care team realized the advanced cancer and unresponsiveness of Carlos's condition, they contemplated the possibility of withdrawing these therapies and initiated a conversation with Carlos's family. Pedro vividly expressed his sorrow upon realising that Carlos's prognosis was grim, regardless of the medical interventions, and he also grappled with the uncertainties surrounding the treatment plan. Conveying the patient's inevitable death and discussing the end‐of‐life care plan with the family brought him a profound sense of sadness and anguish. Pedro represents these emotions as a hazy grey area in the lower‐left corner, with question marks hovering around medications and people's faces. In contrast, in the centre and right of the drawing, he illustrates a vibrant, colourful section that pushes aside the nebulous part, indicated by arrows. This colourful portion represents the positive aspects of patient‐centred end‐of‐life care, symbolising the vital roles played by the medical team, spirituality (depicted as an open book with a flame) and knowledge (represented by upright books). A cross in the drawing signifies the strengthening of professional values when providing compassionate care at life's end. Describing this part of the drawing, Pedro reflects on the peace, fulfilment, and gratification he experienced when recalling that Carlos's family expressed gratitude and found solace in the end‐of‐life care provided. [Color figure can be viewed at wileyonlinelibrary.com]

Students expressed anguish and insecurity when confronted with the uncertainties inherent in end‐of‐life decision‐making. Those uncertainties arose from a lack of theoretical knowledge about the prognosis of severe illnesses and previous experiences with EoL care (Figure 2). They were disturbed and insecure when participating in decisions related to withholding or withdrawing life‐sustaining therapies, such as vasoactive drugs, antibiotics or nasoenteral feeding tubes in a dying patient. They grappled with uncertainty whether these measures would alleviate the patient's suffering or prolong the discomfort of an inevitable death.

**RPS5:** *‘One thing that burdened me a little was that I didn't know what to do. I was a lot like this: “What then?” I wanted something to be done, some decision to be made, but I didn't know what to do. I stayed in that conflict.’*

Sometimes, students also felt guilty when, for instance, they advocated for palliative care and were confronted by supervisors who dismissed them as inexperienced students. In those moments, they said, ‘It looked like I wanted to kill the patient.’

**RPS2:** *‘Because that's what everyone says ‐ that the interns always want to kill the patient. Interns always want to do palliative care and they are always the ones to suggest it. At least now, that I'm going through Intensive Care, it's more evident that we are the ones who suggest palliative care. And then I always have this idea: “Is it because we don't know enough to understand the patient's situation, or is it because we have a different view, the one that views medicine as more than just trying to cure at any cost?” That was the big conflict, you know? Really, I grappled with this suffering and doubt for a long time.’*

All those emotional and cognitive burdens resulting from the death experience, combined with the cognitive burden inherent in the complex decision‐making process, set the context for the central moral dilemma experienced by participants: the decision to establish a meaningful connection with patients or detach from them.

### 3.3 Connecting versus detaching: an upsetting dilemma

Medical students recognised the profound vulnerability and emotional turmoil experienced by individuals facing life‐threatening illnesses. This awareness reminded them that they are not just treating diseases but caring for individuals who are going through an incredibly challenging phase of life. Faced with this vulnerability and emotional turmoil, medical students tend to develop a desire for meaningful connections with their patients. However, this inclination created a complex moral and emotional dynamic. While students acknowledge that forging deeper connections allowed them to deeply understand the patient's needs, preferences and values, thereby promoting patient‐centred EoL care, they also faced significant challenges. These included constant exposure to suffering patients, a clinical culture that advocates emotional detachment as a coping mechanism, systemic constraints such as unsupportive supervisors, and uncertainty about the best course of action for the patient. These factors collectively influenced their tendency to detach, as they navigated the competing demands of their clinical environment and their own emotional well‐being.

**RPS1: ‘** *I feel very lonely, very empty. Because no one in medical school talks about everything I'm telling you today. No one will talk about getting involved with your patient. You can't turn to a professor and start saying that you were involved and felt love in that relationship. We lack this moment for conversation, where we talk about pain about suffering, which is present in everything. For me, medicine confront these every day, daily, constantly, and no one talks about it. This is very sad.’*

**FIGURE 3:**
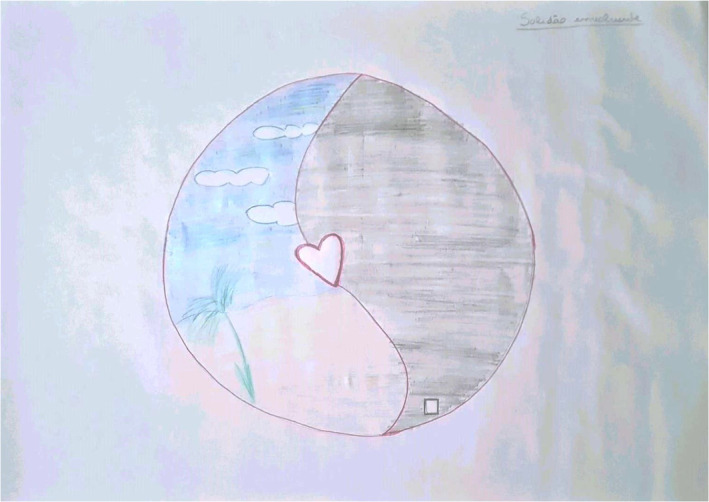
‘Solitude that Involves’ (rich picture 1—RPS1/State University of Campinas, 2022–2023): The student (Barbara—fictitious name) depicts the experience of caring for a 70‐year‐old woman (Tereza, fictitious name) with end‐stage pulmonary fibrosis. Tereza was admitted to the pneumology ward, and Barbara was responsible for evaluating her every morning and discussing her case with the multi‐professional team. Barbara reported having been very involved with Tereza. Tereza shared her life history, sharing her values and beliefs, and talking about the people she cared about the most. Tereza's clinical condition worsened during the hospitalisation, and she died. Barbara represented the experience with the Chinese yin–yang symbol signifying that the good and bad things complement each other. A beach inside the symbol depicts the good feelings that remain at the end. In Barbára's words: ‘It's that moment to watch a sunset on the beach, super involving.’ The white square in the drawing is the loneliness (solitude in the title) that the student feels because she was unable to share what she felt during the experience of connecting with her patient. Barbara reported that she feels like the medical context did not place value on forming a deep connection with the patient, and as a result, she did not feel comfortable sharing her feelings. This lack of connection led her to feel solitude. [Color figure can be viewed at wileyonlinelibrary.com]

While navigating the connection dilemma, students went through whirlwind of emotions. On the one hand, they experienced positive emotions when deepening their relationship with patients and honouring their values, beliefs and wishes. Conversely, students struggled with a contrasting set of feelings when coming into closer contact with their patients' depersonalisation, suffering and eventual death (Figure 3).

**RPS1:** *‘It Involved so many emotions that we would put as supposedly positive ‐ happiness, affection, attention, care, but there was also the element of anguish and suffering because it is tough to see a person with shortness of breath at the end. And you will be missing the person, and that person is going away[…]because I think that, in several situations, things complement each other a lot, no matter how opposite they are, they are very connected.’*

After pondering about the dilemma, students who managed to forge a meaningful connection felt peace and fulfilment. As one student said: ‘It is difficult, but it is good!’ These feelings were accompanied by a transformation. In other words, experiencing death, dealing with the EoL decision‐making, and deciding to connect were a transformative learning experience.

**RPS2:** *‘As I identified the problem, I identified what I was feeling. I identified the problem: people don't understand that they can let others die even if the patients don't have terminal cancer. Then, I was able to take advantage of other opportunities to develop myself as a student and a doctor and to get closer to residents with a similar perspective. For example, accompanying them (the residents) in communicating bad news or when they were, talking to each other about this issue (palliative care in patients who don't have cancer) or when they were discussing other patients' cases during the rounds. Even if I couldn't do it and lead to frustration. It empowered me in some way.’*

### 3.4 Being transformed

Students who possessed the capacity to engage in profound reflection upon their experiences, emotions and the complex moral dilemma they faced in the context of EoL care, were bestowed with a truly transformative learning experience. The transformation began once students engaged with the dilemma, evolved as they delved into a critical reflection on their personal and professional trajectories, and culminated as students incorporated new skills, values and attitudes into their personal and professional lives.

**RPS4** *: ‘I was thinking about giving up (medical school). I don't know why we are afraid of crying. Afraid to get involved with our patients. We're taught that we shouldn't cry (in medical school) and shouldn't get involved. So, we end up creating a barrier. After that experience (participation in EoL care), when I take care of a patient, I will first try to learn the patient's story. Get to know the things that matter or don't matter to them at that moment, identify those who hold importance in their life, understand the core values of their life, and acknowledge how they prefer to receive care.’*

At a personal level, students reflected on their own death and life's meaning. They realised that death would happen to all human beings, including them, which led them to contemplate aspects such as where, with whom and how they would prefer to pass through the dying process. This reflection was accompanied by students trying to answer the question, ‘How am I going to live my life?’ They elaborate on life's meaning—what they believe is right, good and beautiful—and in which direction their life should go—if they want to have children, what kind of citizen they want to be, and whether medicine is the right profession for them.

**RPS3:** *‘Fear of wasting my time just studying medicine and reading medical books, do you understand? Like, Man, I'm wasting my time. I'm not doing personal things I should be doing. Life is one, time is running out, and I'm just learning about the pharmacology receptor.’*

After those reflections, students allocated more time to activities outside of their medical studies. These activities included engaging in sports, travelling, enjoying the company of their family or pursuing personal projects.

**RPS2:** *‘I think I can do what I like, alongside medicine. I have been thinking about it for a while‐ writing books, studying bioethics, writing fiction books that cause reflections on this, novels for people to reflect on things.’*

**FIGURE 4:**
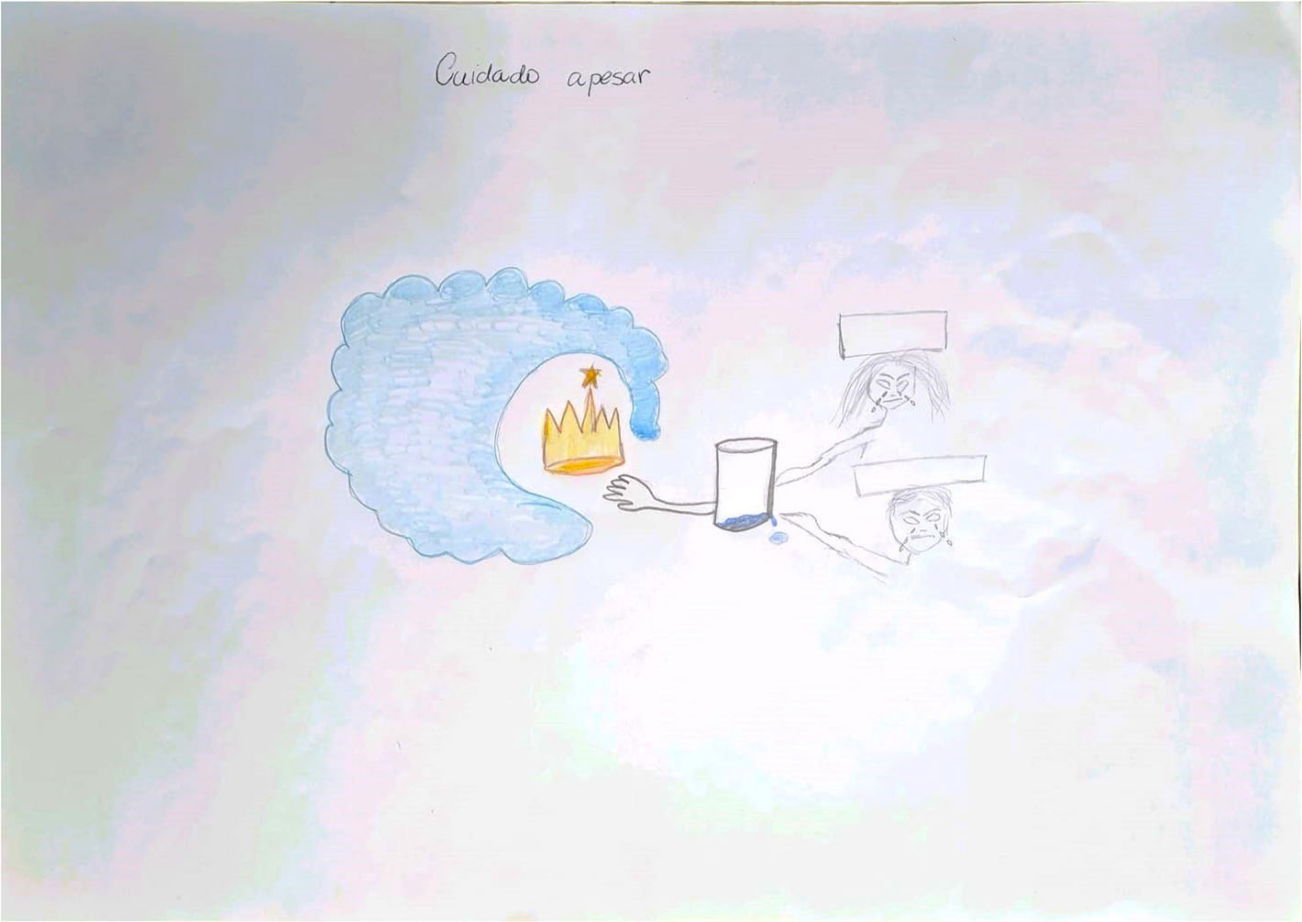
‘Caring despite of’ (rich picture 11—RPS11/State University of Campinas, 2022–2023): The student (Jorge—fictitious name) portrays a situation where he cared for a patient named Lucas (fictious name), a 21‐year‐old man with cirrhosis because of an autoimmune hepatic disease, at the gastroenterology ward. Lucas had multiple nodules of hepatic carcinoma, contraindicating liver transplantation, which limits the therapeutic possibilities. Jorge struggled to communicate the gravity of Lucas's condition to both Lucas and his mother, who remained hopeful. Resident doctors labelled Lucas's mother as overprotective and uncommunicative, discouraging Jorge from getting closer to the patient and his mother. Jorge says that he was very distressed by the situation and frustrated at not being able to get support from resident doctors. As Lucas's condition worsened, the care team sought help from palliative care specialists, which allowed Jorge to participate in a care plan focussed on alleviating suffering. After a few weeks, Lucas died. This experience profoundly impacted Jorge. He learned to value individuals for who they are, regardless of preconceived notions. Jorge was initially sad and frustrated, but his prevailing emotion was hope. After the experience, he began to delve into getting to know people, intending to become a more compassionate and patient‐centred health‐care professional in the future. The title’ caring despite of’ refers to Jorge's commitment to providing care, even without support from resident doctors. He represented his dedication with his hand at the centre of the drawing. The cloud in the illustration symbolizes Lucas's mother, who referred to him as ‘my little king’, which is why a crown represents him. Jorge draws himself as a half‐empty glass to represent his distress about the situation. On the right side of the image, the scalpel‐wielding hands of the resident doctors poke this half‐empty glass, which Jorge made to represent the harassment he suffered. [Color figure can be viewed at wileyonlinelibrary.com]

At a professional level, students reflected on the kind of doctor they aspire to become. This reflection involved contemplating their core professional values, thinking about whether they should distance themselves from their patients or intensify the connection with them and deciding which specialty to follow (Figure 4). These decisions encompassed aspects like choosing a specialty that might involve more or less exposure to situations involving EoL care and death.

**RPS8** *: ‘One thing I reflected on during that case was that when I believe someone is dying, I should act according to the wishes of the person who is dying. So, that's one thing that Juliana's case (patients' fictitious name) made me understand — that getting close to my patient is not a bad thing. After her case, every time I showed affection or stayed close to someone, it did not harm me.’ (the case happened months before the interview, and the student had opportunities to participate in end‐of‐life care for other patients)*

After contemplating the type of doctor they wanted to become and deciding to connect with their patients, students reported changing the way they perceived their profession and acquiring new paramount values, skills and attitudes. They strengthened values related to patient‐centred practice, such as a profound sense of dedication, zeal and respect for patients' autonomy and values. This commitment to patient‐centred care was mirrored in their development of essential skills, such as the ability to deliver empathetic and sensitive difficult news to patients and to compose patient‐centred medical records that acknowledge the individual's uniqueness. Furthermore, these reflective exercises kindled within students a resolute moral courage. This moral courage enabled them to step forward as advocates for their patients during medical rounds, standing up for their patients' rights and best interests. In the context of EoL decisions, students demonstrated a strong determination to fight for their patients' wishes, ensuring that the individuals under their care received the dignified and patient‐centred treatment they deserved. Importantly, the opportunity to express their viewpoints and advocate for their patients while making sense of these clinical dilemmas significantly contributed to their perceived transformation. By feeling connected to their patients and ensuring they had a ‘good death’, students felt that their actions raised a sense of fulfilment in carrying out their profession effectively.

**RPS9** *:‘We learn a lot about disease and treatment. That's very ingrained in me. If I see a patient, I always think about what electrolyte imbalance they might have. Whether I can or cannot replace sodium or potassium. How do I treat this or that disease. And I studied her (the patient) illness. Because that's how we learn to be doctors. After her case, I changed. I went there, talked to her about what she liked, and showed affection. And that's not something we learn in medical school. The faculty finds it strange; some supervisors even judge, find it odd. So, I think this case helped me realize that assisting the patient, caring for them, won't always be solely through disease‐focused techniques and treatments. We have to look at the patient and discern what truly is in their best interest, according to their own desires and needs, and that's something we don't learn in medical school.’*

## 4 DISCUSSION

Our study sheds light on the emotional and cognitive challenges inherent in medical students' moral dilemma when engaging in EoL care—the tension between the imperative to establish genuine connections with patients and the prevailing culture of emotional detachment. This clash between what is learned and what is experienced, between what is desired and what is allowed generates, as expected, identity dissonance.^26^ Although emotionally taxing, and even painful, depending on the context, this dissonance may also offer an opportunity for growth and transformation.^42^, ^43^ In this discussion, delving into Mesirow's concept of transformative learning,^44^ we draw parallels between his theory and the transformative journey of medical students grappling with the decision to connect with or detach from patients.

Mesirow's transformative learning theory^44^ highlights the importance of critical reflection on lived experiences to transform individual perspectives and beliefs.^44^, ^45^ According to Mesirow, transformative learning is ‘learning that transforms problematic frames of reference—sets of fixed assumptions and expectations (habits of mind, meaning perspectives, mindsets)—to make them more inclusive, discriminating, open, reflective, and emotionally able to change.’^44^ Transformative learning begins with a disorientating dilemma, which happens when the person confronts a particular event or experience that challenges their deep‐rooted beliefs.^45^, ^46^ Beliefs, defined as convictions, ideas or values that individuals hold over an extended period, shape their worldview and may, consequently, resist change.^47^ The disorienting dilemma may spontaneously prompt critical reflection when students rigorously scrutinise their existing beliefs and assumptions, potentially altering their understanding of the world.^48^ Questioning and challenging a current understanding of the world often results in emotional discomfort, which serves as a starting point for self‐examination.^49^, ^50^ When first conceptualised, the transformative learning theory mainly focussed on the cognitive development of students; however, it has become clear that such transformative experiences may also impact on students emotional and social development, including their identity development.

In the context of EoL care, the disorienting (moral) dilemma occurs when students feel the urge to connect with patients but must navigate a culture of emotional detachment. The pressure to conform to established norms within the medical community often places students in a position where they must choose between fitting in or staying true to their compassionate inclinations. This clash creates internal turmoil, leading students to question their suitability for the medical profession and their ability to adhere to its norms without compromising their core values. They must decide between aligning with this culture and fulfilling the expectation of supervisors or speaking up and taking the risk of being perceived as ‘outsiders’ or ‘naïve’. Being perceived as ‘outsiders’ threatens students' professional identity development, which means that students need support and reassurance from their supervisors in order to grow out of this dilemma while keeping faith in the medical profession.

Navigating this moral dilemma also creates opportunities for deeper self‐reflection and growth. For this growth to happen, students must find the moral courage to overcome this identity dissonance and connect with patients, which leads to a reinforcement of their sense of purpose in becoming doctors. Cultivating this moral courage is challenging but fundamental to seize such experiences as opportunities to nurture a professional identity committed to patient‐centred care.^51^ However, when students do not find the moral courage to resist these implicit norms and rules, they internalise a need for emotional detachment, which often goes against their inner disposition to connect.^51^ Suppressing the drive to connect often leads medical students to establish relationships with patients that fall short of what they have envisioned.^52^, ^53^ This is perceived as a failure, and students may find themselves emotionally drained and disillusioned, potentially leading to emotional exhaustion, cynicism and a diminished sense of accomplishment.^54^, ^55^, ^56^ The disheartening outcome is a surrender to the prevalent culture of detachment, perpetuating a cycle that has implications not only for the individual students but also for the broader medical community. Recognising and addressing these challenges is vital for breaking this vicious cycle and fostering a medical culture that truly values the human connection in patient care. We believe that medical students and educators could work together to take advantage of these moments of identity dissonance to nurture transformative learning, scaffold medical students' professional identity development and reflect on (and change) the de‐humanising aspects of the medical culture.

Finding the right level of support to engage in meaningful reflections while experiencing a moral dilemma is crucial to seize its transformative powers. Transformative learning involves dealing with edge emotions.^57^ Only by understanding, regulating and managing their emotional responses, students can better cope with the internal conflicts that arise from the disorienting (moral) dilemmas and optimise their learning and development. Thus, for students to take advantage of the moments of identity dissonance as opportunities for transformative learning, medical educators need to support the development of their emotion regulation strategies and foster a more inclusive and empathetic educational environment, ultimately enhancing students' resilience and commitment to patient‐centred care.

### 4.1 Practical implications

Drawing from our research, we highlight practical implications by examining two critical domains. First, we will discuss the importance of teaching emotional regulation strategies as a foundational skill for both personal growth and effective patient‐centred care. Next, we delve into the transformative potential of educators sharing their own experiences to cultivate trust, inclusivity and active engagement. Together, these practical considerations may contribute to a more inclusive, just and transformative educational experience within the realm of EoL care.

*Educators should discuss emotional regulation strategies* – Faculty members should support students in reflecting on the prevalent culture of emotional detachment. One effective approach involves exploring Gross's four primary mechanisms of emotional regulation^58^: selection modification, attention deployment, cognitive reappraisal and response modulation. These mechanisms are universal and an integral part of our strategies to cope with our emotional existence, including clinical experiences.^58^ In selection modification, individuals actively modulate the way they interact with their context to modulate and achieve their preferred emotional state,^59^ like a surgeon playing music inside the operating room. Attention deployment redirects focus within a given situation, which has the power to create or remove space for certain emotional states.^58^ For instance, palliative care specialists move from the frustration of being unable to cure to the opportunity of offering comfort and peace. Cognitive reappraisal pertains to post‐event cognitive reframing, when individuals reassess a situation to change its emotional meaning and, consequently, future responses.^58^ For instance, doctors are able to de‐escalate angry conversation by understanding that patients are suffering. Finally, response modulation encompasses directly adjusting psychological, emotional and behavioural responses to the situation.^58^ For example, when doctors are taking care of a patient in an urgent situation who has had inadmissible racist behaviour. Discussing these mechanisms not only equips students and faculty with a shared vocabulary for emotions but also emphasises the ineffectiveness of suppressing emotions.^60^ It is noteworthy that Gross's research suggests persistent and habitual emotional suppression can lead to increased stress and emotional tension over the long term.^58^ Hence, we believe that by delving into the realm of emotional regulation, educators empower students on their journey of transformative learning, enabling them to nurture meaningful patient connections and engage in patient‐centred care while also safeguarding against the risk of burnout in the long term.

*Educators should share their own meaningful and emotional experiences* – Students may struggle to express their thoughts and feelings even in open and safe spaces. By sharing impactful EoL care experiences and embracing their vulnerability, educators can serve as a potent catalyst for authentic, open and democratic dialogue.^61^ This participatory approach may transform the learning environment into an egalitarian space.^62^ In this reciprocal exchange, educators not only instruct but also learn from their students, creating a dynamic atmosphere that promotes critical consciousness and empowers all participants to actively shape their educational journey.^62^

### 4.2 Limitations

Our research has some limitations. First, the study was conducted at a single medical school in Brazil and cultural factors significantly impact emotional and moral reactions, which might limit the transferability of our findings to other cultural contexts. Additionally, the level of autonomy granted to medical students, which can vary in different contexts, may also yield different results. Second, the lack of longitudinal follow‐up limits the insights regarding students' identity development. Finally, the translation of the results into English might result in some loss of nuances and meanings.

## 5 CONCLUSION

Connecting with patients during EoL care is an emotional process that transcends the clinical realm, challenges and stands in contrast to the prevalent medical culture, and transforms medical students' trajectories toward becoming doctors.

According to the medical educator, lecturer and writer Rachel N. Remen, ‘The meaning of our work lies in its human connections. Finding meaning requires us to bring more of ourselves to our work than is often our custom. This is no easy transformation, because many of us are products of a training that actively discouraged this sort of presence. I emerged from my training with the firm view that a genuine human connection with my patients was unprofessional. But the courage to connect in this way may be the key to finding the deepest meaning and satisfaction in our work.’^63^ By embracing the profound challenge of forging genuine connections in the face of institutional detachment, medical students awaken the latent richness within the practice of medicine, revealing that the true essence of the profession lies in the enduring connections that transcend the boundaries of illness and resonate with our shared humanity.

## AUTHOR CONTRIBUTIONS

**Diego Lima Ribeiro:** Writing–original draft; writing–review and editing; formal analysis; investigation; conceptualization; methodology; data curation. **Daniele Sacardo:** Conceptualization; methodology; data curation; investigation; project administration. **Grazyna Drzazga:** Conceptualization; methodology; writing–original draft; formal analysis; data curation. **Marco Antonio de Carvalho‐Filho:** Conceptualization; investigation; writing–original draft; writing–review and editing; methodology; formal analysis; data curation; supervision; project administration.

## CONFLICT OF INTEREST STATEMENT

The authors declare no conflict of interest.

## ETHICS STATEMENT

The authors received ethical approval for the study from the Research Ethics Committee of the State University of Campinas (CAAE: 55505121.2.0000.5404).

## Supporting information


**Appendix S1.** Supporting Information.

## Data Availability

The data that support the findings of this study are available on request from the corresponding author. The data are not publicly available due to privacy or ethical restrictions.
