# Supplementary material for: Connect or detach: A transformative experience for medical students in end‐of‐life care
Source: Med Educ. 2024 Sep 24;59(4):395–408. doi: 10.1111/medu.15545 (PMC11906276; doi:10.1111/medu.15545)
Supplement: Supplementary file 1 — Appendix S1. Supporting Information. [file MEDU-59-395-s001.docx]

**Appendix 1: Rich Pictures session script and Interview Guide to Follow Rich Picture Generation**

**Rich Pictures session script**

In the realm of end-of-life care, healthcare professionals often face complex decisions related to life-sustaining treatments. These decisions can be emotionally intense and morally challenging. When discussing such situations, two key terms come into play: 'withholding' and 'withdrawing' life-sustaining therapies. 'Withholding' treatment involves not escalating therapies, such as maintaining current treatment levels even as a terminally ill patient's condition worsens. 'Withdrawing' therapy, on the other hand, refers to discontinuing a treatment initially meant to sustain life, such as suspending mechanical ventilation. These decisions are not only complex but also profoundly impactful on healthcare professionals, including medical students who are in the process of developing their ethical and professional identities. In this study, we aim to explore the moral dilemmas that often arise in these situations and investigate their effects on the professional development of medical students.

Moral dilemmas are questions we face every day in which we must decide about issues that involve and interfere with our freedom of choice and the freedom of others. Our choices often pivot between the necessity of adhering to established norms and the aspiration to uphold personal values and ideals of conduct, which we not only impose upon ourselves but also anticipate others to acknowledge and follow or, at the very least, accept. Consequently, we find ourselves navigating situations that unveil profound uncertainties and doubts concerning the optimal course of action, where none of the available options may seem entirely satisfying, yet the imperative to choose the best course remains.

To explore this situation, we would like you to draw a picture representing a relevant experience in which you participated in a decision process about withholding/withdrawing life-sustaining therapies from a patient in which you experienced moral dilemmas. It would be interesting if the drawing captured the complexity of the situation, as well as the emotions involved and the possible conflicts. After you finish, I would like to talk about it. I would also like to record this interview, so I would like to make it clear before starting the interview that you can choose not to answer some of the questions but still answer others. Furthermore, this interview contains some questions that ask for your personal data that will only be used for research purposes, maintaining the anonymity and confidentiality of the information.

What is a "Rich Picture":

- It is a form of visual language; that is, it communicates something with as few words as possible.

- It is a way of capturing everything you know about a situation without worrying about the structure or rational analysis of the facts. It represents a situation rather than a problem and allows you to go back, remember, and look at the entire situation simultaneously. It is a starting point for dealing with complexity or uncertainty in all its diversity.

- It can describe things, ideas, people, and connections – it can represent any of these items, or all of them at the same time, when related to the situation you decided to represent.

- It contains a representation of subjective elements of the situation – characteristics, feelings, conflicts, prejudices, things said and unsaid.

**Interview Guide to Follow Rich Picture Generation**

1) Could you share the story behind the drawing? Feel free to tell it however you want; I won't interrupt you.

2) Could you provide a detailed explanation of the elements, symbols, or themes in your drawing? What inspired your choices and the overall composition?

3) Now, I would like to ask you a few questions related to what you drew:

- What were the internal and external conflicts you experienced and represented in this picture?

- How do/did you feel about this experience?

- What do/did you do when you felt that way?

- How does/did this experience influence your professional life?

- How does/did this experience influence your personal life?

4) Now, please share your thoughts and feelings about the entire experience, encompassing the drawing exercise and our interview. Is there anything specific that stood out to you or that you found particularly meaningful?

5) Thank you very much for participating in the research. Is there anything else you would like to add?
